# Trend, geographical distribution, and determinants of modern contraceptive use among married reproductive-age women, based on the 2000, 2005, 2011, and 2016 Ethiopian demographic and health survey

**DOI:** 10.1186/s12905-023-02789-z

**Published:** 2023-11-27

**Authors:** Teshome Demis Nimani, Zinabu Bekele Tadese, Eyob Eshete Tadese, Fikadu Wake Butta

**Affiliations:** 1 Department of epidemiology and biostatistics, School of Public Health College of medicine and Health Science, Haramaya University, Harar, Ethiopia; 2 Department of Health Informatics, School of Public Health, College of Medicine and Health Science, Samara University, Samara, Ethiopia; 3 Department of nursing, College of medicine and Health Science, Mettu University, Mettu, Ethiopia; 4 Department of Health Informatics, School of Public Health College of medicine and Health Science, Mettu University, Mettu, Ethiopia

**Keywords:** Trend, Modern contraceptive use, Determinants, Geographical variation, Married women, DHS, Ethiopia

## Abstract

**Background:**

The most common family planning method is modern contraception. It is a cost-effective way to reduce maternal and neonatal morbidity and mortality and enable women to make informed choices about their reproductive and sexual health. The trend of modern contraceptive utilization has shown drastic change in Ethiopia, and identifying the major factors contributing to such a drastic change is vital to improving plans and strategies for family planning programs. Therefore, this study analyzed the trend, geographical distribution, and determinants of modern contraceptive use among married reproductive-age women in Ethiopia.

**Method:**

This study used secondary data from the EDHS 2000–2016, collected from a population-based cross-sectional study by the Central Statistical Agency, focusing on married reproductive-age women aged 15–49. The study analyzed the modern contraceptive use trends through descriptive analyses conducted in three phases: 2000–2005, 2005–2011, and 2011–2016. The study utilized bivariable and multivariable logistic regression analyses to identify determinant factors, with significant variables declared using a *P*-value of 0.05 and an adjusted OR with 95% confidence interval. Analysis was conducted using STATA.14 and R. Spatial analysis was done using ArcGIS version 10.8 and SatScan^™^ version 9.6.

**Result:**

A weighted total of 33,478 women are included in the study, with a mean age of 31.4 years (8.6 SD). There was a significant increase in the trend of modern contraceptive use among married women over the study period, from 2000 to 2016, from 7.2% to 2000 to 15.7% in 2005, to 30% in 2011, and to 39.5% in 2016. The maximum increase was seen in the second phase (2005–2011), with a 14.3% increase. Factors like age of respondents, educational status, religion, residence, region, wealth index, number of living children, husbands’ desire to have more children, and media exposure were found to be predictors for modern contraceptive utilization.

**Conclusion:**

The prevalence of modern contraceptive use is below 50%, and there is also evidence of wide geographical variation in modern contraceptive use in Ethiopia. Thus, policymakers, high institutions, and other stakeholders must work collaboratively with the government in order to improve awareness about modern contraceptive use.

## Introduction

Family planning (FP) is the ability of individuals or couples to anticipate and attain their desired number of children, spacing, and timing of their births [1]. Most common family planning methods are modern contraception, which includes female sterilization, male sterilization, the contraceptive pill, intrauterine contraceptive devices, injectable, implants, condoms, diaphragms, contraceptive foam, and contraceptive jelly, lactation amenorrhea methods (LAM), standard days methods (SDM), country-specific modern methods, and respondent-mentioned other modern contraceptive methods (including cervical caps, contraceptive sponges, and others), but does not include abortions and menstrual regulation [2].

Modern contraceptives are a cost-effective way to reduce maternal and neonatal morbidity and mortality, and they create opportunities for women to make informed choices about their reproductive and sexual health, thus enabling them to pursue educational advances and careers [3, 4]. Modern family planning service in Ethiopia started to implement by Family Guidance Association (FGAE), which established in 1966 [5] but showed few signs of expansion for a long period of time. After 1980, the Ministry of Health expanded its family planning services with support programs by UNFPA and other stakeholders. Due to the adoption of the population policy numerous local and international partners in family planning have worked with the government in addressing FP programs and services [6].

In 1996, the Ministry of Health released Guidelines for Family Planning Services in Ethiopia to support health providers and managers as well as expand and ensure quality family planning services in the country [5]. The Government of Ethiopia and NGOs have expanded community-based distribution, social marketing, and work-based services in addition to the preexisting facility-based and outreach family planning services since 2002. Moreover, in the last decade, to increase family planning utilization, integration and linkage between family planning services and HIV/AIDS care, along with maternal and other reproductive health services, have been emphasized in guidelines and strategic documents [5].

Globally, the trends of modern contraceptive utilization have increased slightly, from 54% to 1990 to 57.4% in 2015. While in Africa, the trends of modern contraceptive utilization have increased a little from 23.6% to 2008 to 28.5% in 2015, they continue to be low in sub-Saharan Africa [7].

According to the Ethiopian mini-demographic health survey, the 2019 report indicates that the contraceptive prevalence rate was 41%, but the 2015/16 Ethiopian HSTP planned to achieve a contraceptive prevalence rate of 55% in 2019/20. It shows some increment in the contraceptive prevalence rate, but the increment was not sufficient to achieve the country plan [8].

Globally, nearly 350,000 women die each year, while another 50 million suffer illness and disability from complications of pregnancy and childbirth [9]. Contraceptives help to prevent an estimated 2.7 million infant deaths and the loss of 60 million lives in a year [10]. Family planning in countries with high birth rates has the potential to reduce poverty and hunger and halt 32% of all maternal deaths; nearly 10% of childhood deaths, 90% of abortion-related morbidity and mortality, and 20% of pregnancy-related morbidity and mortality also make a huge contribution to the achievement of universal primary schooling and female empowerment [11, 12]. However, the need for 225 million women to prevent or delay pregnancy is unmet due to significant barriers to obtaining and using modern contraceptive methods [13–15].

Assuring access to all people for their preferred contraceptive methods helps advance several human rights, including the right to life, liberty, freedom of opinion and expression, the right to work and education, and the right to health. For women, especially adolescent girls, the use of contraception prevents pregnancy-related health risks. When births are separated by less than two years, the infant mortality rate is 45% higher than when they are separated by 2–3 years and 60% higher than when they are separated by four or more years apart [16].

The risk of morbidity and mortality associated with pregnancy and childbirth is much higher when pregnancy is unintended, while most pregnancies among young girls in sub-Saharan Africa are unintended or untimed [8].

Along with India, Nigeria, Pakistan, Afghanistan, and the Democratic Republic of the Congo, Ethiopia has contributed about 50% of the maternal deaths [9]. The Ethiopia Demographic Health Surveys of 2000, 2005, 2011, and 2016 gave figures of 871, 673, 676, and 412 and maternal mortality ratios per 100,000 live births, respectively [8]. Different literature shows contraceptive prevalence is still low in Ethiopia but is slowly ascending among women age 15–49, who are at risk of morbidity and mortality related to pregnancy and childbirth.

**Fig. 1:**
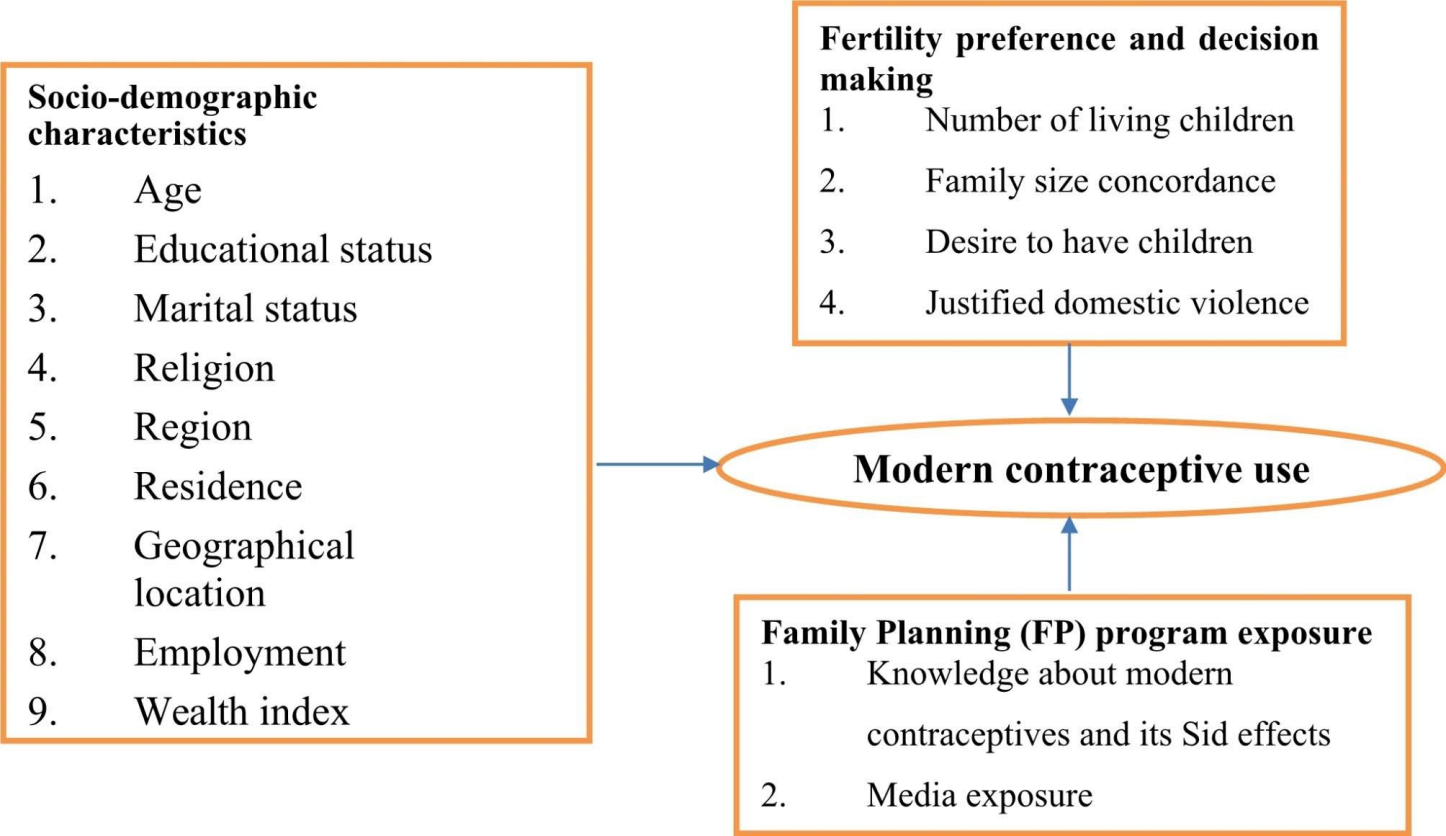
Conceptual framework of modern contraceptive use among married reproductive age women

Factors associated with contraceptive utilization in developing countries, studies showed that the age of the respondent, education status of the respondent, religion, marital status, knowledge about modern contraceptives and side effects, method acceptance by self and partners, geographical location, distance to health service facilities, media exposure, residence, and wealth quintiles [17–22] justified domestic violence [23] displayed according to the literature review [7, 14, 15, 17, 19, 23] displayed the conceptual framework in Fig. 1. Region (low prevalence clusters were seen in Afar, Somalia, and some parts of Gambela Regional State of Ethiopia.) [24], family size concordance, the desire for more children, and the number of living children [22, 25], were factors in the utilization of contraceptives. Similarly, studies in Ethiopia show that age, residence, maternal educational status, couple discussion, perceived partner approval, discussion with health extension workers, perceived cultural acceptance, the desire for more children, monthly income, and numbers of living children were determined by modern contraceptive utilization [15, 24, 26, 27].

Identifying factors associated with changes in contraceptive use among women is vital to improving contraceptive use. The reigning trend in contraceptive use could be due to current changes in demographic composition, including expansion of urbanization, education of girls, and other development activities, or it could be due to changes in contraceptive utilization behavior. Hence, determining the major factors contributing to such a drastic change helps to plan strategies for family planning programs. Therefore, this study is conducted to determine the levels, trends, geographical distribution, and determinants of modern contraceptive use and change among reproductive-aged women in Ethiopia.

## Methods and materials

### Study design, period and setting

The data set for this study was secondary data extracted from the Ethiopia Demographic Health Survey (EDHS) 2000, 2005, 2011, and 2016, which was collected based on a population-based cross-sectional study by the Central Statistical Agency (CSA) from nine regions and two administrative cities.

The study was conducted in Ethiopia (30-40 N and 330-480E), situated at the eastern tip of Africa, which is located at the horn of Africa (one of the tenth largest countries in Africa). The projections for the 2007 population and housing census estimate the population of the nation at 108,805,142 in 2018. In the administrative structure of the country, there are nine regional states and two city administrations subdivided into 68 zones, 817 districts, and 16,253 Keeble’s (the lowest local administrative units of the country).

### Data source and extraction

**Fig. 2:**
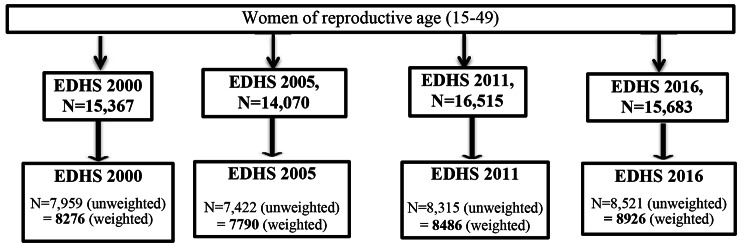
Flow chart for selection of study participants and final sample size of modern contraceptive use among married reproductive age women

The data for this analysis were extracted from all four EDHS data sets, which can be accessed from the DHS website (http://www.dhsprogram.com). The survey is usually conducted at five-year intervals in a country. Ethiopia has undertaken four consecutive DHS surveys: in 2000, 2005, 2011, and 2016, and two minimum DHS surveys in 2014 and 2019. It is a secondary data analysis from a nationwide community-based survey. The data sets were downloaded in STATA format. The necessary data (individual records of women) for all EDHS was cleaned and appended using STATA version 14. In this study, the data is restricted to reproductive-age women (15–49 years of age). Based on these criteria, our sample sizes from the four Ethiopian Demographic and Health Surveys (EDHS) were 8276 women in 2000, 7790 in 2005, 8486 in 2011, and 8926 in 2016 (Fig. 2). The exclusion pregnant at the time of the survey.

### Data management, data processing and analysis methods

The trend in modern contraceptive use was analyzed using descriptive analyses (frequency, percentage, text, figures, and tables), stratified by region, urban-rural residence, and selected socio-demographic characteristics. The trend was examined separately for the periods 2000–2005, 2005–2011, and 2011–2016. Logistic regression analysis was also done to identify the determinants of modern contraceptive use among women of reproductive age using data from the 2016 EDHS. A *P*-value less than 0.25 was considered a cutoff point to select variables during bivariable logistic regression for the final model of multivariable logistic regression. Finally, a *P*-value less than 0.05 and an adjusted OR with the correspondence 95% confidence interval were used to declare the significant variables. All the above-mentioned analyses were conducted using STATA 14. Spatial analysis was done using ArcGIS version 10.8 and SatScan^™^ version 9.6.

### Eligibility criteria

#### Inclusion criteria

Married reproductive-age women (from 15 to 49 years old) are the total eligible women (see Fig. 2).

#### Exclusion criteria

Pregnant women.

### Study variables

#### Dependent variable

Current modern contraceptive use.

#### Independent variables

Socio-demographic Characteristics (Age, Educational status, Marital status, Religion, Region, Residence, Geographical location, Employment, and Wealth index), Fertility preference and decision making (Number of living children, Family size concordance, Desire to have children, and Justified domestic violence), and Family Planning (FP) program exposure (Knowledge about modern contraceptives and its Side effects and Media exposure).

### Operational definition

Utilized Modern contraceptive methods: women who say they use one of the following methods: female sterilization, male sterilization, the contraceptive pill, intrauterine contraceptive device, injectables, implants, condom, diaphragm, contraceptive foam, and contraceptive jelly; lactational amenorrhea method; standard days method; respondent-mentioned other modern contraceptive methods, including cervical caps, contraceptive sponges, and others, but does not include abortions and menstrual regulation.

## Result

### Socio-demographic characteristics

A total of 33,478 women are included in the study, both married and non-pregnant. The mean age of the respondents was 31.4 years About 75.5% of the respondents had no education, while 20.4% had primary education. Across the three EDHS surveys, the proportion of Orthodox Christians showed a decline from 50.0 to 41.1% between 2000 and 2016, while the proportion of Muslims increased from 29.6 to 33.8%. The proportion of women who resided in rural areas remained relatively the same at 88.3% in 2000 and 88.9% in 2005, then decreased to 83.9% in 2016. About 19.2% of the respondents fell into the poorest wealth quintile in 2005 and slightly fell to 18.8% in 2016, whereas 19.4% were classified in the richest quintile in 2005 and slightly increased to 20.8% in 2016. The data is presented in Table 1.

### Fertility preference and decision-making

Concerning family size concordance, the percentage of husbands who wanted more children than their wives declined from 26.9% to 2000 to 17.1% in 2005, rising again to 25.9% in 2016. The percentage of husbands wanting the same number of children as their wives wanted (concordance) rose from 34.3% to 2000 to 39.5% in 2011, and the rest of the data is presented in the Table 1.

### Family planning (FP) program exposure

The proportion of people who heard about family planning on radio has increased from 14.7% to 2000 to 22% in 2016. Most of variables listed in Table 1 showed decreasing changes, when comparing the sample population in year 2000 with 2016.

**Table 1:** Percentage distribution of characteristics of the respondents, 2000, 2005, 2011 and 2016 Ethiopia Demographic and Health Surveys

		EDHS 2000 N = 8276	EDHS 2005 N = 7790	EDHS 2011 N = 8486	EDHS 2016 N = 8926	EDHS 2000–2016 N = 33,478
Variables	Categories	n (%)	n (%)	n (%)	n (%)	N’ (%)
Age	15–19	671 (8.11)	569 (7.31)	603 (7.1)	482 (5.4)	2325 (6.95)
	20–24	1447 (17.49)	1285 (16.5)	1368 (16.12	1375 (15.4	5475 (16.36)
	25–29	1630 (19.7)	1736 (22.28)	2003 (23.6	2030 (22.74)	7398 (22.10)
	30–34	1343 (16.23)	1306 (16.76)	1393 (16.42	1797 (20.13)	5839 (17.44)
	35–39	1285 (15.53)	1166 (14.97)	1354 (15.96)	1459 (16.35)	5265 (15.73)
	40–44	981 (11.85)	887 (11.38)	908 (10.7)	1010 (11.31)	3785 (11.31)
	45–49	918 (11.09)	841 (10.8)	857 (10.1)	774 (8.67)	3390 (10.13)
Educational Status	No education	6877 (83.1)	6107 (78.4)	5694 (67.1)	5597 (62.7)	24275 (72.51)
	Primary	968 (11.7)	1176 (15.1)	2232 (26.3)	2446 (27.4)	6822 (20.38)
	Secondary	381 (4.6)	428 (5.5)	305 (3.6	527 (5.9)	1641 (4.9)
	Higher	50 (0.6)	78 (1.0)	255 (3.0)	357 (4.0)	739 (2.21)
Religion	Orthodox	4138 (50.0)	3599 (46.2)	3859 (45.48)	3695 (41.4)	15292 (45.68)
	Muslim	2450 (29.6)	2485 (31.9)	2692 (31.72)	3017 (33.8)	10643 (31.79)
	Protestant	1316 (15.9)	1433 (18.4)	1723 (20.3)	1999 (22.4)	6471 (19.33)
	Catholic	58 (0.7)	86 (1.1)	68 (0.8)	62 (0.7)	274 (0.82)
	Other	314 (3.8)	187 (2.4)	144 (1.7)	152 (1.7)	797 (2.38)
Region	Tigray	521 (6.3)	467 (6.0)	526 (6.2)	541 (6.06)	2056 (6.14)
	Afar	108 (1.3)	93 (1.2)	93 (1.1)	70 (0.78)	364 (1.09)
	Amhara	2251 (27.2)	2072 (26.6)	2469 (29.1)	2147 (24.05)	8939 (26.7)
	Oromia	3153 (38.1)	2836 (36.4)	3301 (38.9)	3393 (38.01)	12683 (37.88)
	Somali	91 (1.1)	312 (4.0)	187 (2.2)	186 (2.08)	775 (2.31)
	Benishangul	91 (1.1)	78 (1.0)	102 (1.2)	96 (1.08)	367 (1.1)
	SNNPR	1779 (21.5)	1651 (21.2)	1451 (17.1)	1772 (19.85)	6654 (19.87)
	Gambela	25 (0.3)	23 (0.3)	34 (0.4)	24 (0.27)	106 (0.32)
	Harari	17 (0.2)	16 (0.2)	25 (0.3)	15 (0.17)	73 (0.22)
	Addis Ababa	207 (2.5)	203 (2.6)	272 (3.2)	272 (3.05)	953 (2.85)
	Dire Dawa	33 (0.4)	39 (0.5)	25 (0.3)	411 (4.6)	508 (1.52)
Residence	Urban	968 (11.7)	865 (11.1)	1519 (17.9)	1437 (16.1)	4789 (14.31)
	Rural	7308 (88.3)	6925 (88.9)	6967 (82.1)	7489 (83.9)	28689 (85.69)
Wealth index	Poorest	0	1496 (19.2)	1697 (20.0)	1678 (18.8)	4871 (14.55)
	Poorer	0	1589 (20.4)	1748 (20.6)	1785 (20.0)	5122 (15.3)
	Middle	0	1605 (20.6)	1748 (20.6)	1839 (20.6)	5192 (15.51)
	Richer	0	1589 (20.4)	1629 (19.2)	1767 (19.8)	4986 (14.89)
	Richest	0	1511 (19.4)	1663 (19.6)	1857 (20.8)	5031 (15.03)
**Fertility preference and decision-making**						
Number of living children Family size	0	761 (9.2)	569 (7.3)	696 (8.2)	330 (3.7)	2356 (7.04)
	1	1258 (15.2)	1067 (13.7)	1188 (14.0)	1366 (15.3)	4879 (14.57)
	2	1349 (16.3)	1161 (14.9)	1400 (16.5)	1401 (15.7)	5311 (15.86)
	3	1192 (14.4)	1130 (14.5)	1281 (15.1)	1482 (16.6)	5084 (15.19)
	4+	3716 (44.9)	3864 (49.6)	3921 (46.2)	4347 (48.7)	15847 (47.34)
Family size concordance	Both wants the same	2839 (34.3)	2555 (32.8)	3547 (41.8)	3526 (39.5)	12467 (37.24)
	Husband wants more	2226 (26.9)	1332 (17.1)	2037 (24.0)	2312 (25.9)	7907 (23.62)
	Husband want fewer	439 (5.3)	374 (4.8)	721 (8.5)	1241 (13.9)	2775 (8.29)
	Don’t know and missing	2772 (33.5)	3529 (45.3)	2181 (25.7)	1848 (20.7)	10330 (30.86)
Desire for Wants children	Within 2 year	2086 (25.2)	1371 (17.6)	1604 (18.9)	1642 (18.4)	6703 (20.02)
	After 2 + years	2764 (33.4)	2610 (33.5)	3055 (36.0)	3079 (34.5)	11508 (34.38)
	Unsure timing	223 (2.7)	241 (3.1)	178 (2.1)	241 (2.7)	884 (2.64)
	Undecided	248 (3.0)	62 (0.8)	280 (3.3)	446 (5.0)	1037 (3.10)
	Wants no more	2955 (35.7)	3506 (45.0)	3369 (39.7)	3517 (39.4)	13346 (39.86)
**Family Planning (FP) program exposure**						
Do you know about Modern contraceptive	Yes	7076 (85.5)	6824 (87.6)	8257 (97.3)	8819 (98.8)	30976 (92.53)
	No	1200 (14.5)	966 (12.4)	229 (2.7)	107 (1.2)	2502 (7.47)
Heard about FP on Radio	Yes	1217 (14.7)	1994 (25.6)	2580 (30.4)	1964 (22.0)	7754 (23.16)
	No	7059 (85.3)	5796 (74.4)	5906 (69.6)	6962 (78.0)	25724 (76.84)
Heard about FP on TV	Yes	207 (2.5)	514 (6.6)	1197 (14.1)	1232 (13.8)	3149 (9.41)
	No	8069 (97.5)	7276 (93.4)	7289 (85.9)	7694 (86.2)	30329 (90.59)
Read about FP on newspaper and magazine	Yes	157 (1.9)	327 (4.2)	399 (4.7)	277 (3.1)	1160 (3.46)
	No	8119 (98.1)	7463 (95.8)	8087 (95.3)	8649 (96.9)	32318 (96.54)

### Trends of modern contraceptive

**Fig. 3:**
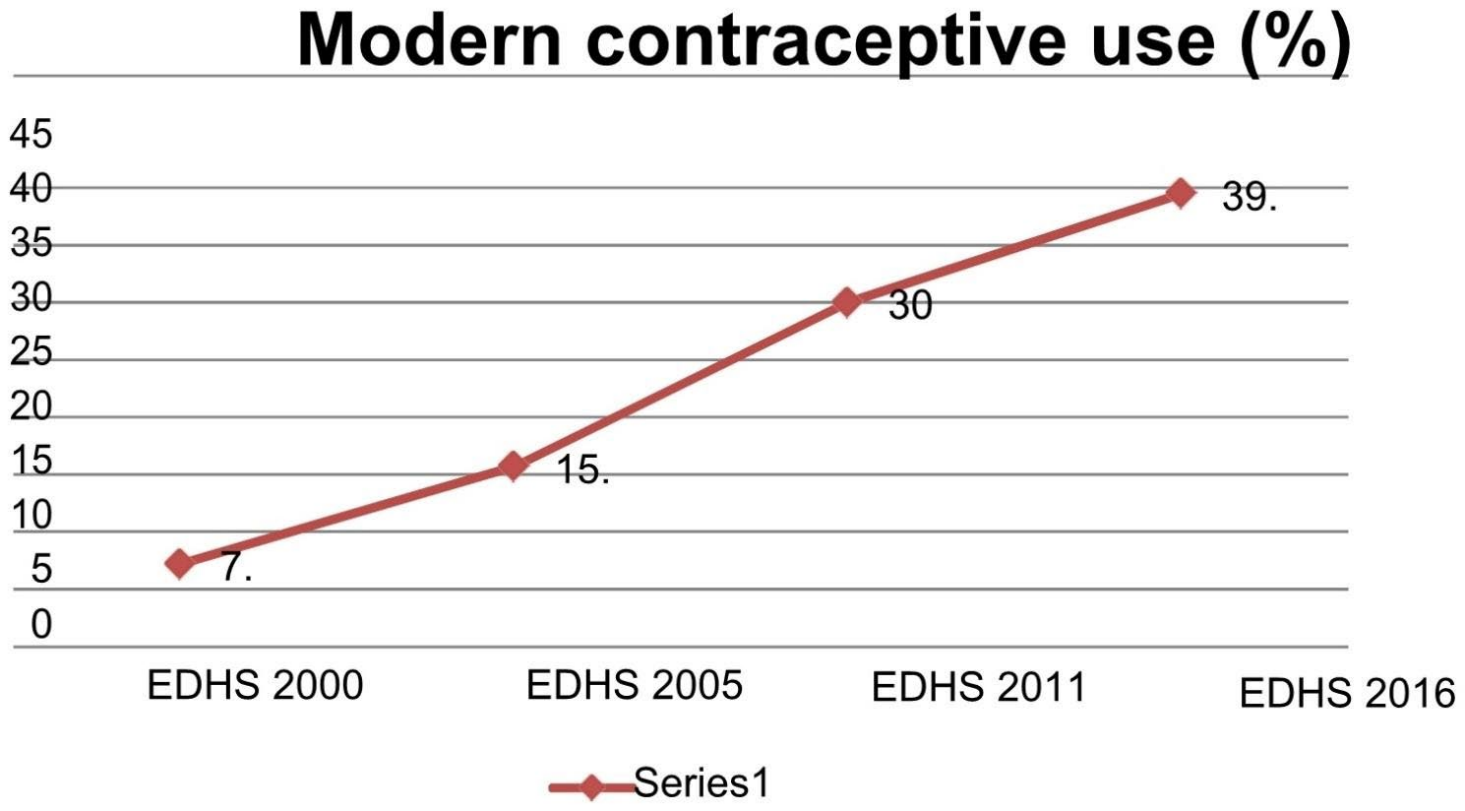
Trends in modern contraceptive use among married reproductive age women in the past 15 years, Ethiopian Demographic and Health Surveys, 2005–2016 (N = 33,478)

The trend of each independent variable with its respective survey year is displayed in Table 2 and graphical in Fig. 3.

**Table 2:** Trend in modern contraceptive use among married reproductive age women by selected Characteristics, 2000, 2005, 2011 and 2016 Ethiopia Demographic and Health Survey

Characteristics	Categories	EDHS 2000	EDHS 2005	EDHS 2011	EDHS 2016	Phase 1	Phase 2	Phase 3
		N = 8276	N = 7790	N = 8486	N = 8926			
Age	15–19	4	4.5	5.8	5.1	0.5	1.3	-0.7
	20–24	14.6	18.9	20	17.9	4.3	1.1	-2.1
	25–29	26.3	26.4	25.8	26.8	0.1	-0.6	1
	30–34	20.4	17.1	19.8	21	-3.3	2.7	1.2
Number of living children Family size	2	16.3	17.6	21.4	18.2	1.3	3.8	-3.2
	3	20.1	16.6	16	15.8	-3.5	-0.6	-0.2
	4+	45.8	47.1	39	40.8	1.3	-8.1	1.8
Family size concordance	Both wants	53.6	45.7	54.2	45.9	-7.9	8.5	-8.3
	Husband wants more	25	15.6	19.6	22.2	-9.4	4	2.6
	Husband wants Fewer	7.8	7.2	10	7.6	-0.6	2.8	-2.4
	Don’t know	13.6	31.5	16.2	24.3	17.9	-15.3	8.1
Desire for Wants children	within 2 year	9.5	6.4	11.3	11.5	-3.1	4.9	0.2
	Wants after 2+	30.1	36.9	40.7	42.2	6.8	3.8	1.5
	Wants, unsure Timing	1.4	1.2	1.4	2	-0.2	0.2	0.6
	Undecided	1.2	0.5	2.5	3.9	-0.7	2	1.4
	Wants no more	0	55	44.1	40.4	-2.8	10.9	-3.7
**Family Planning (FP) program exposure**								
Do you know about modern contraceptive	Yes	7.5	15.5	32.3	44.7	8	16.8	12.4
	No	92.5	84.5	67.7	55.3	-8	16.8	-12.4
Heard about FP on radio	Yes	43.4	47.1	41.6	27.3	3.7	5.5	-14.3
	No	56.6	52.9	58.4	72.7	-3.9	5.5	14.3
Heard about FP on TV	Yes	12.4	19.5	26.6	17.4	7.1	7.1	-8.9
	No	87.6	80.5	75.4	82.6	-7.1	-5.1	7.2
Read about FP on newspaper and magazine	Yes	7.8	12.3	8.1	4.3	4.5	-4.2	-0.2
	No	92.2	87.7	91.9	95.7	-4.5	4.2	3.8
Educational Status	No Education	49.2	55.8	53.5	54.3	-6.6 0	2.3 0	-0.8 0
	Secondary	22.7	17.1	6.9	8.9	5.6	10.2	-2
	Higher	4.1	2.9	6.3	5.6	1.2	-3.4	0.7
Religion	Orthodox	61.2	61.3	54.7	50.5	-0.1	6.6	4.2
	Muslim	24.6	20.2	23	21.2	4.4	-2.8	1.8
	Protestant	12	16.4	20.9	26.8	-4.4	-4.5	-5.9
	Catholic	0.7	0.9	0.8	0.9	-0.2	0.1	-0.1
	Other	1.5	1.2	0.6	0.6	0.3	0.6	0
Region	Tigray	8.8	7	4.8	6.4	1.8	2.2	-1.6
	Afar	1.6	0.5	0.4	0.3	1.1	0.1	0.1
	Amhara	2.8	28.9	34.5	31.5	-26.1	-5.6	3
	Oromia	2.6	34.3	36.1	31.1	-31.7	-1.8	5
	Somali	0.3	0.8	0.3	0.1	-0.5	0.5	0.2
	Benishangul	1.5	0.8	1.2	0.9	0.7	-0.4	0.3
	SNNPR	17.7	18.3	15.2	24.2	-0.6	3.1	-9
	Gambela	0.6	0.4	0.4	0.3	0.2	0	0.1
	Harari	0.7	0.5	0.3	0.2	0.2	0.2	0.1
	Addis Ababa	12.9	7.6	6.5	4.6	5.3	1.1	1.9
	Dire Dawa	1.4	0.9	0.3	0.4	0.5	0.6	-0.1
Residence	Urban	53.7	30.9	32.1	22.2	22.8	-1.2	9.9
	Rural	46.3	69.1	67.9	77.8	-22.8	1.2	-9.9

### Spatial autocorrelation analysis of modern contraceptive use

**Fig. 4:**
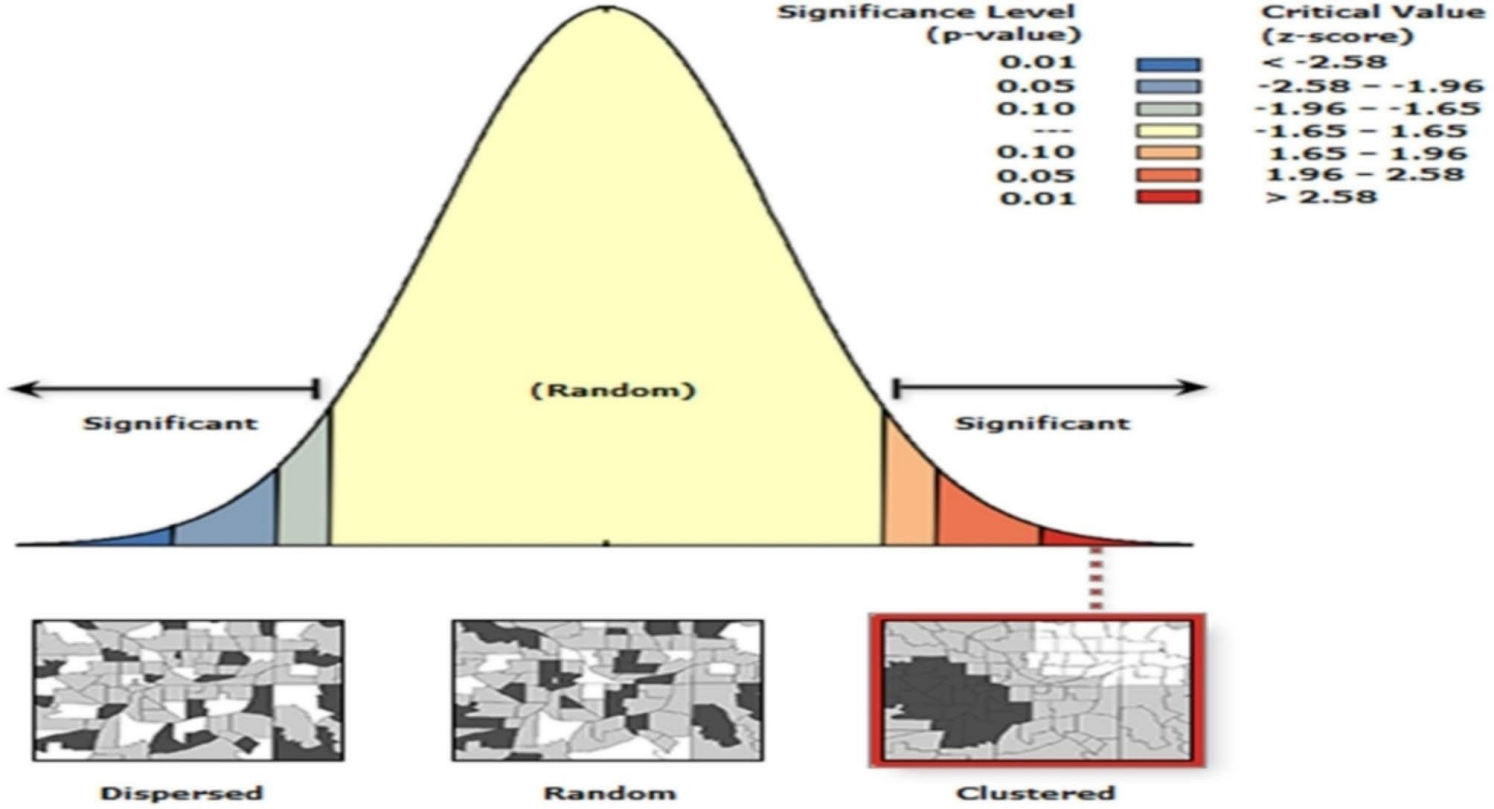
Spatial autocorrelation analysis of modern contraceptive use among reproductive age women in EDHS 2016

When we see Global Moran’s I Summary, it has Moran’s Index = 0.35, Variance = 0.000272, z-score = 21.183917, and *p*-value = 0.000000 displayed in Fig. 4.

### Hotspot analysis of modern contraceptive use

**Fig. 5:**
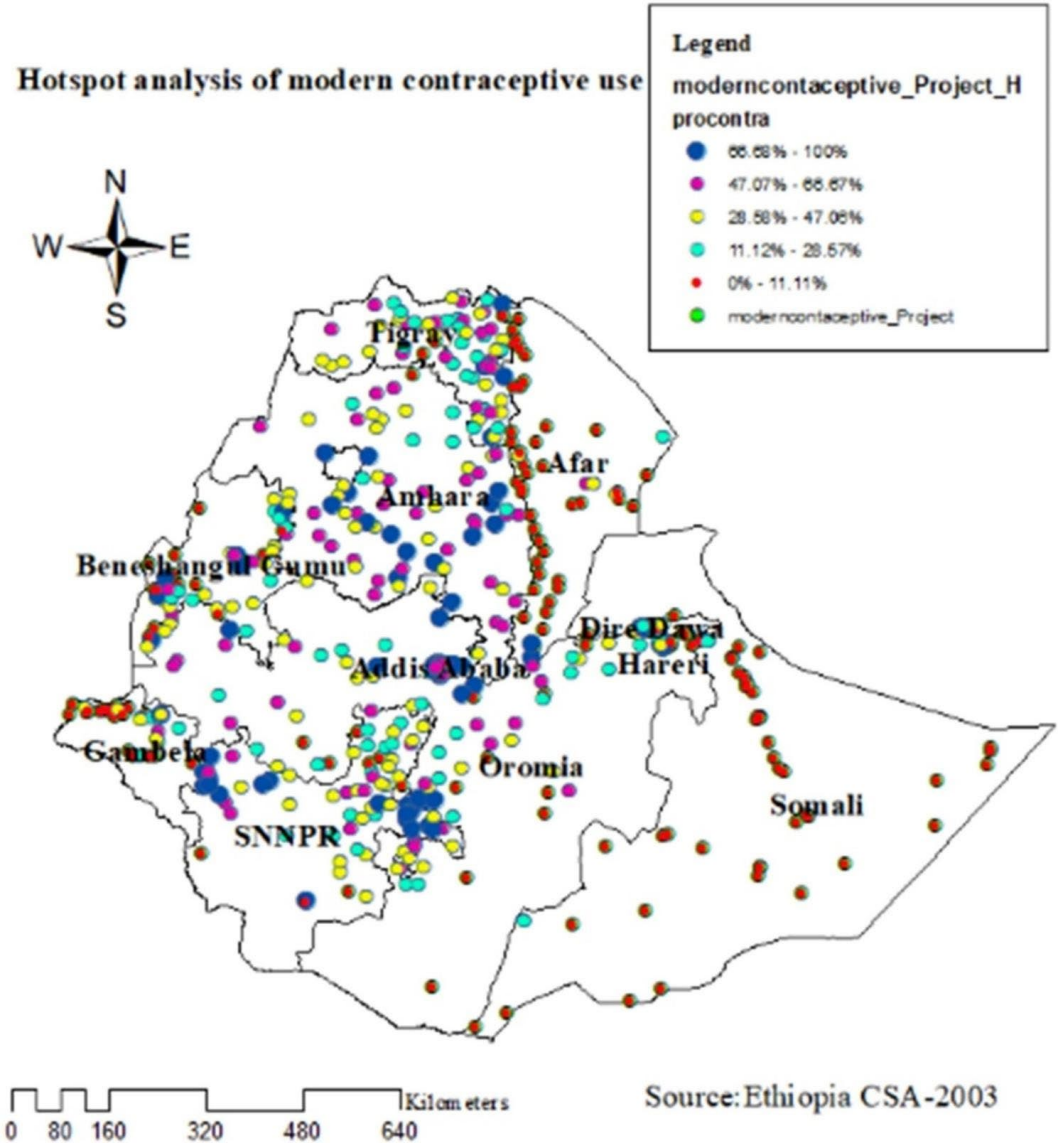
Hot spot analysis of modern contraceptive use among reproductive age women in EDHS 2016

Hot and cold spots analysis point out risk areas of low family planning usage. The hot spot (high risk) regions that is low modern contraceptive usage were detected Somalia, Harari some part of Oromia Afar and Dire Dewa. The hotspot analysis indicates significance high prevalence areas of low modern contraceptive utilization and Z-score increases in both directions which quantify significant low and high low modern contraceptive utilization (see Fig. 5).

### Spatial interpolation for prediction of modern contraceptive use

**Fig. 6:**
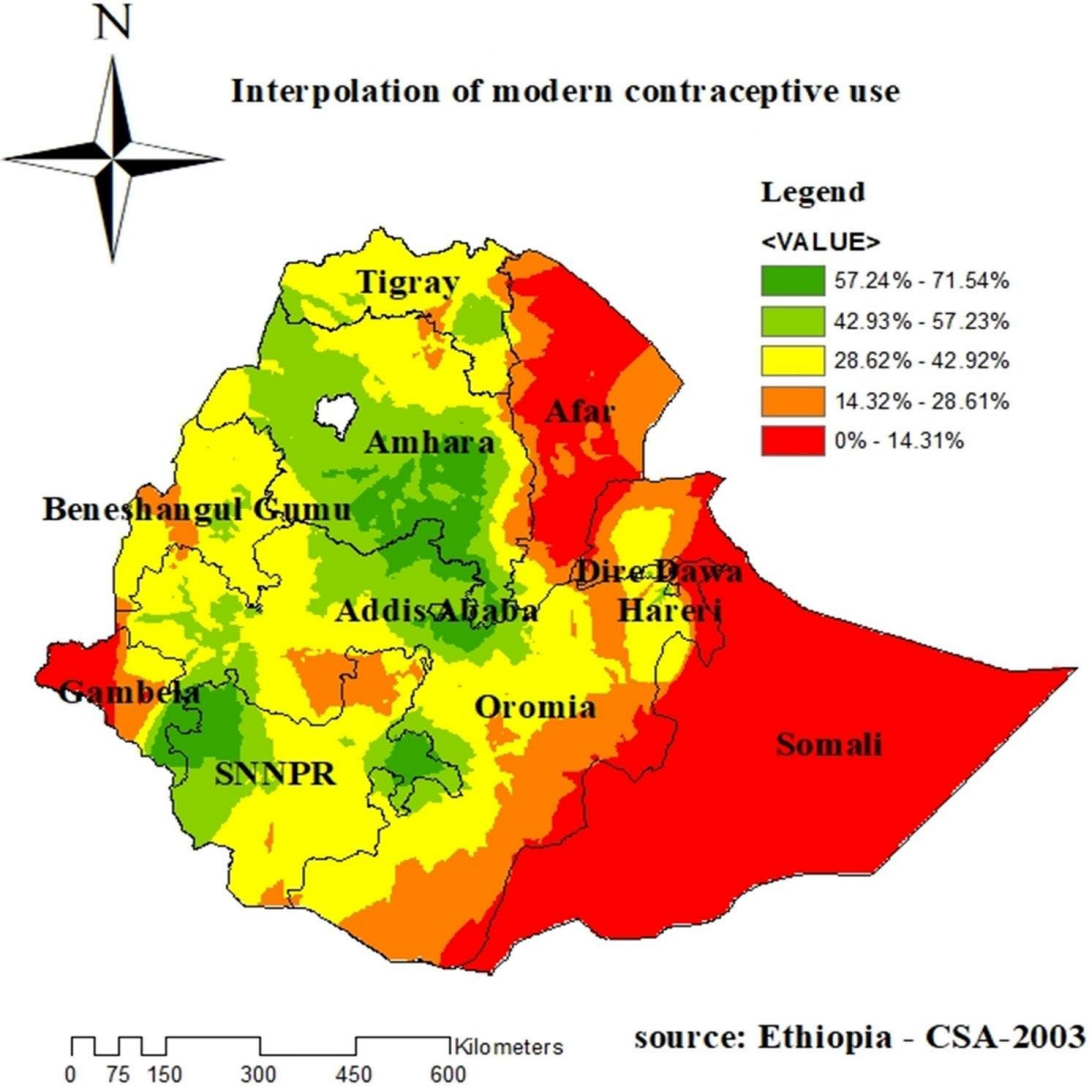
Interpolation of modern contraceptive use among reproductive age women in EDHS 201

The spatial kriging interpolation analysis predicted high-risk regions for low modern contraceptive usage; these are Somalia, Dire Dewa, Afar, and Gambela, and Addis Abeba, Amhara, some parts of Oromia, some parts of SNNP, and some parts of Tigray (see Fig. 6).

### Sat scan analysis of modern contraceptive use

**Fig. 7:**
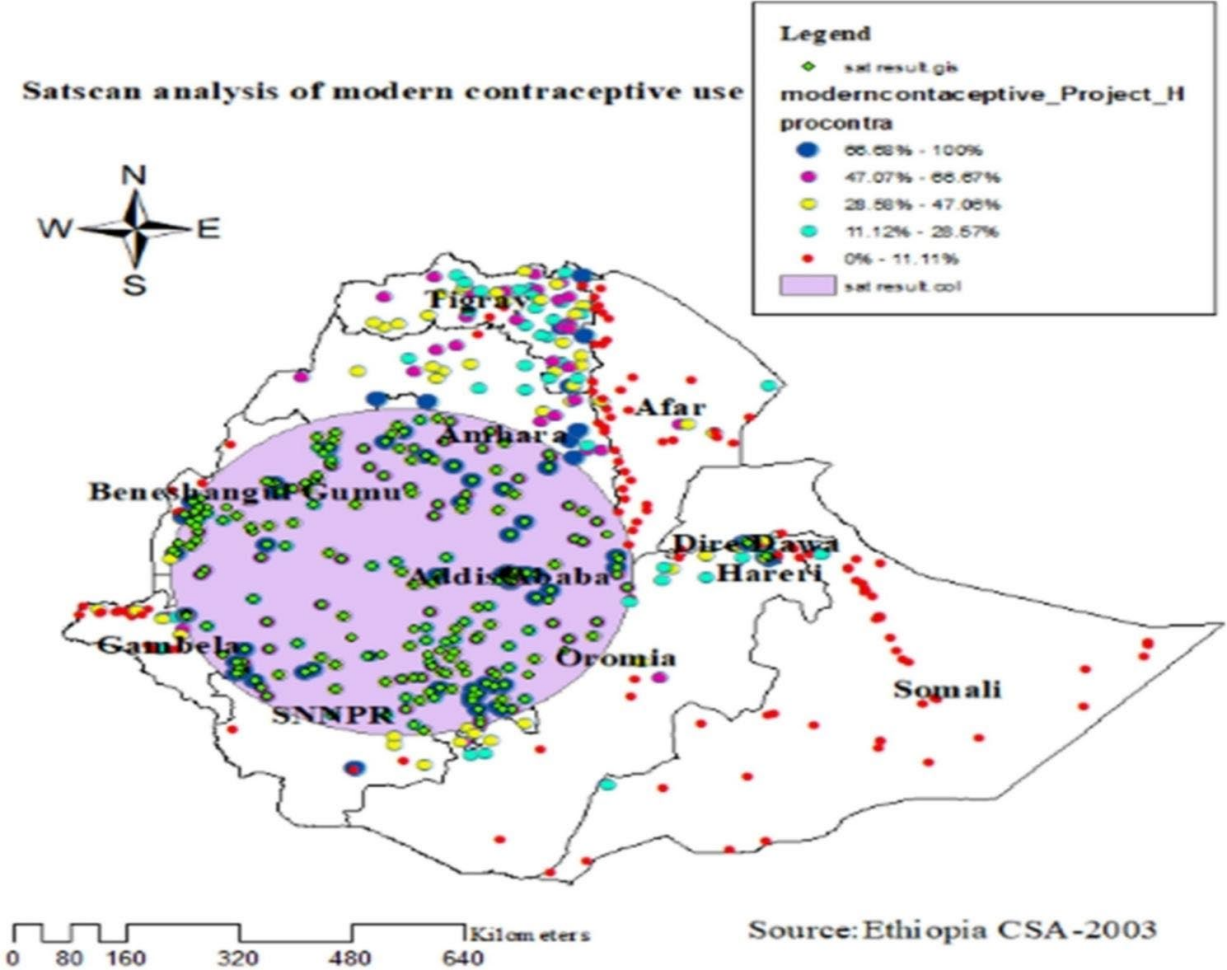
Sat Scan analysis of modern contraceptive use among reproductive age women in EDHS 2016

Spatial scan statistics identified primary (LLR = 230.95, P 0.00000000001) and secondary (LLR = 21.84, P 0.00000049) clusters of modern contraceptive utilization using the maximum spatial circular windows of 25% of the total population (Table 3; Fig. 7). The large primary cluster spatial window encompasses Addis Abeba, the Oromia region, Amhara, and SNNP. It was centered on a relative risk (RR) of 1.95. Married women within the spatial window were 1.95 times more likely to use modern contraceptive methods than married women outside the window.

**Table 3:** Sat Scan analysis of modern contraceptive use in 2016 EDHS data

	Primary cluster	Secondary cluster	Tertiary
Population	3778	210	84
Number of Cases	1733	117	49
Relative risk	1.95	1.68	1.74
Percent of cases in area	45.9	55.7	58.3
Log likelihood Ratio (LLR)	230.95328	21.844014	10.725983
*P*-value	1E-17	0.00000049	0.012

### Factors associated with changes in modern contraceptive use

Bivariate analysis was run in logistic regression to check the association between dependent and independent variables. Accordingly, age 20–34 and 45–49 years, education (illiterate and primary educated), religion (Muslim), region except rural residence, wealth index, and number of living children were identified as determinant factors. Variables that were found to be associated with the outcome variable in the bivariate analysis (*P* = 0.25) were taken to the multivariable analysis. This is basically to compensate for the power of the test since negative findings (that is, *p* > 0.05) may be just because of inadequate power. After adjusting for possible confounding factors, the age of respondents, educational status, religion, residence, region, wealth index, and number of living children were found to be predictors of modern contraceptive utilization. Women of age group greater than 25–29 had 17% [AOR = 1.17:CI 1.001, 1.357] higher utilization compared to women of reproductive age 15–19; women of age 45–49 are 4.5 times more likely to use modern contraceptives when compared to those of age 15–19 [AOR = 4.5: CI 3.603, 5.601]; women of age 40–44 are 2.07 [AOR = 2.07: CI: 1.714, 2.500] times higher modern contraceptive utilization when compared with those aged 15–19; those between 35 and 39 ages had 43% [AOR = 1.43, CI: 1.204, 1.700] increased utilization when compared with those of 15–19 displayed in Table 4.

Women who had two children had 18% (AOR: 0.82, at 95% CI: 0.702, 0.979) lower odds of contraceptive use than women who had four or more children. Likewise, women had 15% (AOR: 0.85, at 95 CI%: 0.731, 0.988) lower odds of contraceptive use if they had 3 children than those who had 4 + children, but those who had 0 children are 1.54 times more likely to use modern contraceptives. When we see media exposure, those who heard about family planning on TV have 66% higher odds of using contraceptives than women who did not hear about family planning on TV, but those who did not hear about family planning on radio are 1.26 times more likely to use contraceptives than those who heard. Compared with both wanting the same children, odds of using contraceptives were 1.26 times (AOR; 1.26, at 95% CI: 1.116, 1.422), 1.22 times (AOR; 1.22, at 95% CI: 1.016, 1.468), and 1.28 times (AOR; 1.28, at 95% CI: 1.139, 1.440) higher among husbands who wanted more, fewer, and didn’t know, respectively. Desire for children has a significant effect on modern contraceptive utilization. When compared with couples who want children within two years, the odds of using contraceptives were 1.39, 1.06, and 12.67 times higher among couples who want children after 2 + years, who want children but are unsure of the timing, and who want no more children, respectively, but couples who have not decided when to have children had 46% lower contraceptive use than those who want children within two years displayed in Table 4.

**Table 4:** Final logistic regression model with crude and adjusted odds ratio using the 2016 Ethiopia Demographic and Health Survey (n = 8926)

Variables	Category	Contraceptive use (%)	COR (95% CI)	AOR (95% CI)	*p*-value
Age	15–19	5.1	1	1	
	20–24	17.9	0.69 [0.56,0.86]	0.93 [0.807, 1.07]	0.339
	25–29	26.8	0.68 [0.55,0.83]	1.17 [1.0, 1.36] *	0.048
	30–34	21	0.85 [0.69, 1.04]	1.32 [1.115, 1.55] *	< 0.001
	35–39	15.2	1.03 [0.83, 1.27]	1.43 [1.204, 1.70] *	< 0.001
	40–44	9.8	1.15 [0.92, 1.44]	2.07 [1.714, 2.50] *	< 0.001
	45–49	4.2	2.48 [1.92, 3.20]	4.50 [3.603, 5.60] *	< 0.001
Education status	No education	54.3	2.45 [1.97, 3.04]	1.48 [1.067, 2.06] *	0.019
	Primary	31.2	1.55 [1.24, 1.94]	1.25 [1.125, 1.37] *	< 0.001
	Secondary	8.9	0.86 [0.66, 1.13]	1.66 [1.520, 1.81] *	< 0.001
	Higher education	5.6	1	1	
Religion	Orthodox	50.5	1	1	
	Muslim	21.2	2.83 [2.55, 3.14]	1.66 [1.52,1.81] *	< 0.001
	Protestant	26.8	1.04 [0.94, 1.16]	1.25 [1.12,1.38] *	< 0.001
	Catholic	0.9	0.98 [0.59, 1.60]	1.48 [1.07,2.06] *	0.019
	Other	0.6	5.69 [3.58, 9.04]	3.47 [2.11,5.72] *	< 0.001
Region	Tigray	6.4	1	1	
	Afar	0.3	4.28 [2.18, 8.38]	1.59 [0.752,3.346]	0.226
	Amhara	31.5	0.59 [0.49, 0.71]	0.51 [0.42,0.63] *	< 0.001
	Oromia	31.1	1.32 [1.10, 1.58]	1.01[0.815,1.256]	0.915
	Somali	0.1	36.8 [14.1, 95.9]	9.78 [3.65,26.2] *	< 0.001
	Benishangul	0.9	1.31 [0.83, 2.05]	0.99 [0.602,1.631]	0.979
	SNNPR	24.2	0.74 [0.61, 0.89]	0.74 [0.58,0.94] *	0.013
	Gambela	0.3	0.98 [0.44, 2.21]	1.17 [0.475,2.880]	0.731
	Harari	0.2	1.22 [0.48, 3.07]	0.96 [0.341,2.689]	0.936
	Addis Ababa	4.6	0.54 [0.41, 0.72]	0.89 [0.640,1.231]	0.475
	Dire Dawa	0.4	1.37 [0.71, 2.66]	1.16 [0.564,2.398]	0.683
Residence	Urban	22.2	1	1	
	Rural	77.8	2.09 [1.87, 2.35]	1.74 [1.412, 2.14] *	< 0.001
Wealth index	Poorest	10.6	3.77 [3.25, 4.36]	1.75 [1.745, 2.67] *	< 0.001
	Poorer	18.1	1.93 [1.69, 2.20]	1.11 [1.113, 1.67] *	0.003
	Middle	21.4	1.54 [1.35, 1.75]	0.89 [0.893, 1.331]	0.397
	Richer	22.5	1.33 [1.16, 1.51]	0.81 [0.814, 1.205]	0.92
	Richest	27.4	1	1	
Number of living children	0	6.6	0.86 [0.52, 0.76]	1.54 [1.2,1.98] *	< 0.001
	1	18.5	0.54 [0.50, 0.74]	0.96 [0.797,1.164]	0.698
	2	18.2	0.52 [0.61, 0.89]	0.82 [0.70, 0.98] *	0.028
	3	15.8	0.63 [0.98, 1.39]	0.85 [0.73,0.99] *	0.035
	4+	40.8	1	1	
Family size concordance	Both wants the same	45.9	1	1	
	Husband wants more	22.2	1.66 [1.49,1.8] *	1.26 [1.12,1.42] *	< 0.001
	Husband want fewer	7.6	1.23 [1.04,1.46]	1.22 [1.02,1.47] *	0.034
	Don’t know	24.3	1.55 [1.39,1.73]	1.28 [1.14,1.44] *	< 0.001
Desire for children	Wants within 2 year	11.5	1	1	
	Wants after 2 + years	42.2	1.69 [1.27,2.24]	1.39 [1.02,1.90] *	0.036
	Wants, unsure timing	2	1.54 [1.25,1.90]	**1.06 [0.841,1.342]**	0.611
	Undecided	3.9	0.94 [0.86,1.03]	0.64 [0.57,0.72] *	< 0.001
	Wants no more	40.4	2.25 [1.61,3.13]	12.67 [4.49,35.79] *	< 0.001
Heard about FP on radio	Yes	27.3	1	1	
	No	72.7	1.66 [1.49,1.83]	1.26 [1.11,1.42] *	< 0.001
Heard about FP on TV	Yes	17.4	1	1	
	No	82.6	1.64 [1.46,1.85]	0.66 [0.543,0.80] *	< 0.001
Read about FP	Yes	4.3	1	1	
	No	95.7	1.74 [1.62,1.87]	1.11 [0.84,1.47]	0.464

Note: * = Statistically significant, COR = Crude Odd Ratio and AOR = Adjusted Odd Ratio

## Discussion

The study was aimed at assessing trends in modern contraceptive utilization among married, non-pregnant women of reproductive age. The study revealed that the trend of contraceptive utilization in Ethiopia is increasing, from 7.2% to 2000 to 39.5% in 2016. This trend is consistent with the Ethiopian Demographic Health Survey, which was 6% in 2000 and 35% in 2016 [22]. The reason for the increase may be due to increased women’s awareness about contraceptives and the increased work of the government and other stakeholders towards family planning services [28]. Based on analysis of the 2016 EDHS, modern contraceptive use showed a significant association with the socio-demographic characteristics of married women. The odds of using contraception were 2 and 4.5 times higher among 40–44 and 45–49-year-old reproductive women, respectively, similar to observations from a study conducted in Nigeria [17]. The odds of modern contraception utilization increased with the age of women; that shows a 17%, 32%, and 43% increase among 25–29, 30–34, and 35-39-year-old married women, respectively, when compared with 15–19 years of age. This could be partly explained by the fact that as young women grow older, many changes come together, such as cognitive maturation, awareness, and other psychosocial skill developments; thus, they are more likely than younger women to consider contraceptive options. Women who reported not being educated and having primary and secondary education were significantly more likely to have modern contraceptives than those educated at a higher level. The study revealed significant differences in contraceptive use across different religions. Muslim women have the highest odds of contraceptive use, followed by Catholics and Protestants, compared with Orthodox Christians. Some studies indicate different observations [24]. However, the role of religion in contraceptive use is not well studied in Africa. This analysis showed an inverse relationship between the number of living children and modern contraceptive use among married women. Women who didn’t have any children had 54% increases in contraceptive utilization when compared with those who had more than four children. This is supported by a previous study [25]. The number of living children has an effect on contraceptive utilization; women who have more than four children are more likely to use modern contraceptives than those who have three or fewer children, which is a similar finding to a study done in Ethiopia and Bangladesh. This might be due to the fact that when the number of surviving children increases, couples want to restrict their birth [22, 23].

Women whose husbands want to have more children than they do have lower odds of contraceptive use compared with women whose husbands want a similar number of children. Likewise, they have lower odds of contraceptive use if they do not know how many children their husbands want to have. Other similar studies also found similar result [14, 22]. This might be due increase of women decision-making power towards different issue including contraceptive use.

In this study, an increase in the proportion of women who heard of family planning in TV had a significant positive contribution to the increase of modern contraceptive use during the last decade. A similar finding was reported in Tanzania [29]. Media such as TV remain a powerful tool to reach a large number of women and provide information regarding modern contraceptives but surprisingly women who heard radio is less likely to use modern this might be audio visual is better than audio only or user of radio small in number. So their need be intensified efforts on awareness creation on modern contraceptives through media especially in TV. As expected, the study found that the odds of contraceptive use are higher when women want a child later or want to limit births, compared with women who want a child soon.

The spatial distribution of for modern contraceptive utilization across the Ethiopia region showed significant variation and clustering. The Global Moran’s I value 0.35 (*p* value = 0.000) indicated that there was significant clustering of modern contraceptive use in Ethiopia. The spatial distribution analysis also indicates significant variations of modern contraceptive utilization across Ethiopia.

The utilization of data from the Ethiopian Demographic and Health survey provided an opportunity for generalization of our findings due to a large sample size and statistical power to make conclusions on modern contraceptive use and asses the geographical variation of modern contraceptive utilization.

Although this study highlighted important findings to support family planning programs in Ethiopia, we could not examine the effect of some important variables such as use of health insurance, partner awareness of contraceptive use, number of sexual partners, religion and family planning services availability as well quality. These variables have been reported to influence modern contraceptive use.

## Conclusion

Although modern contraceptive use among young married women has shown an increase over the last 15 years in Ethiopia, we need to work hard since the contraceptive prevalence is below 50%. Wealth index, age, religion, women’s education, family size concordance, fertility preference, hearing about family planning on TV, number of living children, region, and residence were significantly associated with married reproductive-age women’s modern contraceptive use. There is evidence of wide geographical variation in modern contraceptive use in Ethiopia. Low-prevalence clusters were located in Afar, Somalia, and some parts of Gambela Regional State, Ethiopia. Since the geographical variation of the conceptive use is high in Ethiopia, every concerned stakeholder needs to work together to make the variation balanced, and interventions to reduce the unmet need for family planning by considering the prevalence and spatial distribution should be considered. Strengthening community-based and school-based family planning programs are strategies to maintain young women’s contraceptive use and advance it further.

## Data Availability

The datasets generated and/or analyzed during the current study are available in the Ethiopian demographic and health repository, http://dhsprogram.com/data/available-datasets.cfm.

## Abbreviations

AOR: Adjusted odds ratio
AIDS: Acquired Immune Deficiency Syndrome
CI: Confidence Interval
DHS: Demographic and Health Survey
EDHS: Ethiopian Demographic and Health Survey
FGAE: Family Planning Guidance Association of Ethiopia
FP: Family Planning
FPW: Family Planning Worker
HIV: Human Immune Virus
HSTP: Health Service Transformation Plan
LAM: Lactational Amenorrhea
LLR: Log Likelihood Ratio
NGO: Non-governmental organizations
OR: Odds Ratio
SDM: Standard Day Method
TV: Television
UNFPA: United Nation Population Fund
